# Study protocol of the DUCATI-study: a randomized controlled trial investigating the optimal common channel length in laparoscopic gastric bypass for morbid obese patients

**DOI:** 10.1186/s40608-015-0059-z

**Published:** 2015-07-15

**Authors:** Ralph P.M. Gadiot, Brechtje A. Grotenhuis, L. Ulas Biter, Martin Dunkelgrun, Hans J.J. Zengerink, Pierre B.G.M. Feskens, Guido H.H. Mannaerts

**Affiliations:** Department of Surgery, Sint Franciscus Gasthuis, Rotterdam, The Netherlands; Department of Surgery, Erasmus MC, Rotterdam, The Netherlands; Department of Bariatric Surgery, Lievensberg Ziekenhuis, Bergen op Zoom, The Netherlands

**Keywords:** Morbid obesity, Bariatric surgery, Laparoscopic Roux -Y gastric bypass, Common channel

## Abstract

**Background:**

Morbid obesity has become one of the most frequent chronic medical disorders in Western countries, affecting 1.5-2 % of the Dutch population. Currently, the laparoscopic Roux-Y gastric bypass is considered to be the most effective bariatric treatment option for morbid obesity as it results in adequate weight loss and a significant decrease in comorbidity. Although this technique has been applied for years, the optimal lengths of the three bowel limbs (alimentary limb, biliopancreatic limb, and common channel) in order to achieve maximal percentage excess weight loss with minimal side effects (i.e. malabsorption symptoms), are unknown. As ‘normal’ sized gastric bypasses achieve an average of 60 − 80 % excess weight loss after one year, one could hypothesize that afferent limb lengths should be longer in order to reduce the common channel length, thereby improving outcome in terms of excess weight loss.

The aim of the current study is to investigate the effect of the length of the common channel in gastric bypass surgery for morbid obesity. In this randomized controlled trial the very long Roux limb gastric bypass will be compared to the standard gastric bypass, in order to conclude which option is the optimal therapeutic strategy in the morbidly obese patient.

**Methods/design:**

In this multicentre trial patients will be randomized either to a very long Roux limb gastric bypass with a fixed common channel length of 100 cm, or to a standard gastric bypass with a variable common channel length.

The primary objective is to evaluate whether the very long Roux limb gastric bypass is superior in terms of percentage excess weight loss after one year follow-up compared to the standard gastric bypass.

Secondary endpoints are quality-of-life, cure /improvement of obesity related comorbidity, complications, malnutrition, re-admission rate, and re-operation rate.

**Discussion:**

We hypothesize that our proposed distal LRYGB will provide for improved results concerning % EWL with an acceptable rate of (metabolic) complications.

Our main point of interest is to determine if the distal LRYGB is a superior alternative to standard LRYGB in terms of percentage excess weight loss and to put more focus on the role of the common channel. Therefore we will perform this randomized controlled trial comparing both techniques, with % EWL as a primary outcome.

**Trial registration:**

CCMO registration number: NL43951.101.13 and Netherlands Trial Registry number: NTR4466.

## Background

Obesity is a global problem in Western societies. The World Health Organization (WHO) states that currently obesity is the most serious chronic health problem worldwide [1]. In some western industrial countries over 50 % of the population is overweight. The mortality risk is doubled for women with BMI of >40 kg/m^2^ and tripled for men. Moreover, the earlier obesity occurs with regard to age, the stronger the rise in mortality risk [2].

Treating morbid obesity (defined as a BMI > 40 kg/m^2^) by non-surgical methods has only resulted in minor effects. Surgical treatment has gained widespread acceptance in recent decades due to its superior outcomes in weight loss [3–5]. In 1992 the WHO stated that bariatric surgery is the only effective treatment to achieve long lasting weight loss [1]. Furthermore, bariatric surgery has profound effects on obesity-related co-morbidities, such as type 2 diabetes mellitus, hypertension and sleep apnea, [6]. Bariatric surgery is also safer than conservative treatment for obesity in terms of a lower lifelong mortality risk caused by morbid obesity [7].

Surgical interventions for morbid obesity can be divided into two categories: interventions that cause food malabsorption and interventions that cause food restriction. Laparoscopic Roux-en-Y gastric bypass (LRYGB) has been acknowledged to be safe and effective for the treatment of morbid obesity, and is globally considered the golden standard surgical technique [1, 7–9].

After one year follow-up, LRYGB results in an average of 60–80 % excess weight loss (% EWL) with reduction in comorbidities. However, a partial weight regain can be seen during long-term follow-up [10].

**Fig. 1:**
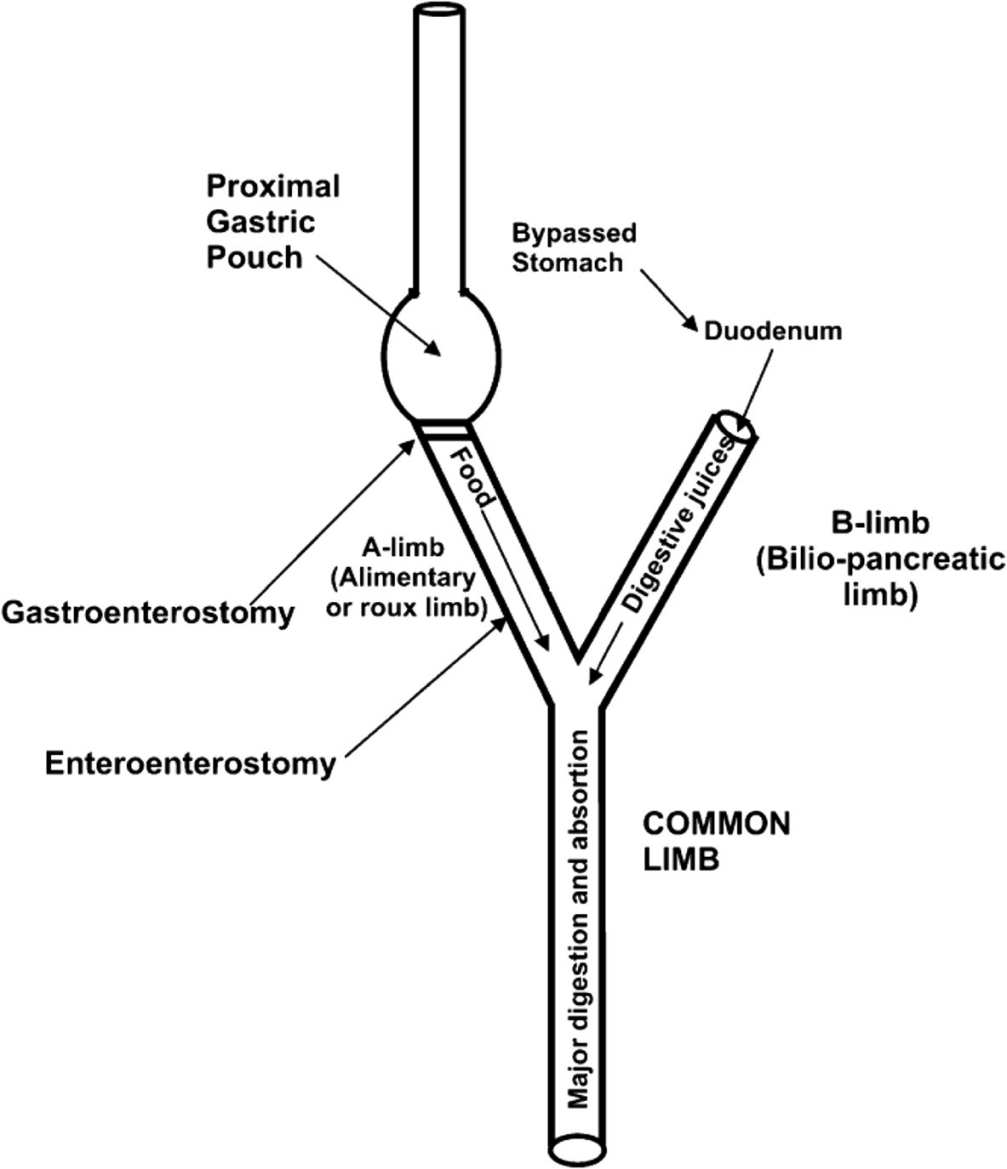
Schematic overview of the limbs of the LRYGB

Consensus does exist on the need for a small calibrated gastric pouch in the LRYGB in order to achieve improved % EWL results. However, to date little attention has been given to determine the optimal lengths of the various intestinal limbs that comprise the operation (the alimentary or ‘Roux’ limb and the biliopancreatic limb or afferent limb) (see Fig. 1). These limb-lengths are important as they determine the amount of weight loss and the incidence of adverse events related to malnutrition and malabsorption.

No consensus has been reached on the optimal lengths of the limbs. In an internet-based study performed by Madan et al. on preferred technique of 215 bariatric surgeons, all of whom are members of the American Society for Metabolic and Bariatric Surgery, showed a wide range of the AL and BL limbs lengths (average 110 [range 35–250]cm for AL, and 48 [range 10–250]cm for BL) [11].

The common channel (where the food arriving through the alimentary limb (AL) is mixed with gastric, bilary, and pancreatic fluids coming from the biliopancreatic limb (BL) (Fig. 1), absorbs most of the nutrients and preserves enterohepatic circulation of bile salts and fat [12]. Some absorption of amino acids and glucose may occur in the AL as well, through the saliva and succus which may digest proteins and carbohydrates. In the BL, as the digestive fluids pass through its mucosa, reabsorption of digestive components such as lipases may also occur, which decrease the digestive potential of the fluid reaching the food in the common channel [12].

In the specific population of super-obese patients it has been suggested that an increase in malabsorbtion may be established by creating a longer AL, resulting in an increase in weight loss in this selected group of patients [13, 14].

A review of the available literature on different limb lengths in LRYGB identified four randomized controlled trials (RCT).

It has been concluded that the currently available literature supports the notion that a longer AL (at least 150 cm) may be associated with a modest weight loss in the short-term for superobese patients, but without a significant impact on patients with BMI < 50 [13, 14]. Limitations of the available literature include the heterogeneity of studies, small study populations, and lack of standardization. Furthermore, the length of the common channel was not accounted for in these studies.

Nevertheless, there is evidence that the degree of malabsorption after gastric bypass is influenced mainly by the length of the common channel [15]. Therefore it has been suggested that good quality studies that focus on the impact of the length of the common channel on weight loss are urgently needed [14].

When searching for the optimal lengths of the bypass limbs, it is known that one cannot lengthen the alimentary limb without facing metabolic complications. Complications associated with biliopancreatic diversion and very long-limb RYGB are mostly due to a too short common channel [16]. Kalfarentzos et al. [17] found that 100 cm common channel in biliopancreatic diversion with RYGB and long limbs is a safe length for achieving satisfactory % EWL without major nutritional deficiencies.

In 2006 Nelson et al. [18] described a “very, very long limb” Roux-en-Y gastric bypass in a series of super obese patients in which a 100 cm CC was created, with a 60 cm BL and a “very, very” long Roux or AL of 400 to 500 cm. In this study 82 % of patients lost >50 % of excess body weight. Nine (4 %) patients required re-operation due to protein/calorie malnutrition in this group by proximal relocation of the enteroenterostomy with symptom resolution in all patients.

Recently Thurnheer et al. [19] published on a so called distal very long RYGB with an average CC of 76 cm (±7 cm), BL of 79 cm (±14 cm) and variable AL lengths of 604 cm (±99 cm). Average EWL in this population of 355 patients was >74 % up to 5 years after the operation and failure rate was relatively low (<6 %). The incidence of severe malnutrition states was relatively low (1.7 %) and the complication rate (4.5 % major and 10.4 % minor complications) was comparable to the literature.

We hypothesized that distal LRYGB as stated in the present proposal will have similar results in terms of % EWL as the distal very long RYGB described by Thurnheer on the % EWL after 1 year (69.7 %; SD 18.3), this will be used to calculate group sizes for this study. The proposed technique was first described by the group of Sarr [20] and termed very, very long limb Roux-en-Y gastric bypass (VVLL-RYGB). We feel that very long Roux limb gastric bypass (VLRL-RYGB) describes the proposed technique more clearly and will therefore be used in this article.

Data required for the calculation of group sizes was not reliable and therefore the data from the study by Thurnheer [19] will be used for this purpose.

In conclusion, currently there is no consensus about the optimal length of the alimentary, biliopancreatic limb and common channel for achieving best weight loss results, and more studies are needed to determine the role of the common channel in LRYGB.

We hypothesize that VLRL-RYGB (with standardised common channel length of 100 cm) will result in a better weight loss result, in terms of % EWL after one year when compared to standard LRYGB.

## Methods

### Study objective

To determine if the very long Roux limb gastric bypass is superior to standard gastric bypass as a surgical treatment for morbid obesity in terms of percentage excess weight loss after one year.

### Study design

The DUCATI-trial is a multi centre, randomized controlled, trial. The study compares very long Roux limb gastric bypass and standard laparoscopic Roux - Y gastric bypass. Patients will be randomly allocated to A) VLRL-RYGB or B) standard LRYGB and will be followed for a period of at least 1 year. The study will be performed in a clinical and outpatient setting with regular visits at 2, 6, and 12 months post intervention.

Randomisation is stratified for the participating centres. Randomisation takes place in the operation room and is single blinded.

### Patient selection

Study subjects are selected from a clinical population of the Sint Franciscus Gasthuis, Rotterdam, The Netherlands and the Lievensberg hospital, Bergen op Zoom, The Netherlands. All morbidly obese patients who have had no prior bariatric surgery and who are eligible for primary laparoscopic gastric bypass surgery are candidates for the trial.

Inclusion criteria are:

- Age 18–60 years
- BMI > 40, or >35 kg/m2 with obesity related co-morbidity
- Psychological screening excluding psychiatric and psychological disorders
- Informed consent and willing to enter the follow up program after the operation.

Exclusion criteria are:

- Prior bariatric surgery
- Prior major abdominal surgery (like colonic resection, septic abdomen, aorta surgery, or other procedures with a high risk of intra-abdominal adhesions, which might jeopardise the possibility of performing a VLRL-RYGB, standard LRYGB
- ASA (American Society for Anesthesiologists) classification ≥ IV
- Pregnant women
- Endocrine causes, alcohol or drug abuse
- Severe concomitant disease (carcinomas, neurodegenerative disorders or other disorders presently representing being considered exclusion criteria for bariatric surgery )
- The inability of reading/understanding and filling out questionnaires
- VLRL-RYGB or LYRGB is technically not possible as will be determined by the surgeon during surgery.

### Study questions and outcome measures

Primary endpoint: Is the VLRL-RYGB superior to standard LRYGB in terms of percentage excess weight loss after one year?

Excess weight (kg) will be calculated with the formula EW = AW-IW (actual weight- ideal weight), IW = 22 × L^2^ (L = length in meters). The amount of weight loss will be expressed as percentage excess weight loss (%EWL), and calculated with the formula % EWL = (pre-operative BMI – current BMI) / (pre-operative BMI-25) × 100 %.

Secondary endpoint: what is the effect of the interventions on quality-of-life (QOL), cure /improvement of obesity related co-morbidity, complications, malnutrition side effects, re-admission rate, and re-operation rate?

The following outcome measures will be analysed to answer these secondary questions:

Patient’s health-related quality of life (QoL) objectified by the MOS Short Form 36 (SF 36), Gastro-Intestinal Quality of Life Index, and Obesity related Quality of life the Moorehead-Ardelt II questionnaires and the Bariatric Analysis and Reporting Outcome System (BAROS) score.

DM-II, hypertension, hypercholesterolemia, GERD, OSAS and joint-pain will be scored as worsened, same, improved or cured at follow-up visits.

Operating time, mean hospital stay, intra-operative and post-operative morbidity, and in-hospital mortality. Morbidity is defined as reoperations, reinterventions, re-admissions and serious adverse events. Morbidity is classified as major (anastomotic leakage, major peroperative blood loss due to splenic or vascular hemorrhage, pulmonary embolism, intra-abdominal abscess and intra-abdominal hematoma) or minor (wound infection, urinary tract infection and anastomotic stenosis) complications. For analysis the Clavien-Dindo classification will be used [21]. Moreover, the rate of extra outpatient and ER visits due to complaints are recorded.

Biochemical and hormonal values following VLRL-RYGB and standard LRYGB will be evaluated by laboratory testing of the following parameters: Vitamin B1, B6, B12, D, folic acid, HbA1C, ferritin, iron, transferrin, cholesterol, HDL-cholesterol, LDL-cholesterol, triglyceride, calcium, magnesium, albumin, Apo-B, Zinc, homocysteine, parathomone.

### Surgical interventions

After reviewing the literature we hypothesized that a standardized common channel length of 100 cm should be safe to reduce major nutritional deficiencies, while remaining a long alimentary limb. To ensure a degree of comparability between VLRL-RYGB and standard LRYGB, the BL was set at a fixed length of 60 cm.

Two different approaches for anastomosis are known, being the linear and the circular gastro-enterostomy stapling techniques, which are *both allowed* in this study, as it does not seem to affect the primary outcome measure; the percentage excess weight loss result (% EWL) [22]. The only prerequisite is that the diameter of the gastro-enterostomy anastomosis will be comparable. As the applied circular stapler anastomosis is 25 mm, the linear stapled anastomosis will need to be 25 mm in diameter as well. The type of technique linear or circular will be registered.

#### Very long Roux limb gastric bypass

Technique (linear gastro-enterostomy stapling technique):

Approximately 6 cm below the angle of His a calibrated gastric pouch is created using a 34 Fr gastric tube. After creation of the pouch the omentum may be split vertically to ensure safe mobilization of the small intestine if necessary.

The common channel is measured 100 cm from the ileocoecal junction and marked with a temporary suture.

Next, the gastro-enterostomy is created 60 cm from the point of Treitz, followed by creation of the entero-enterostomy between the small intestine proximal from the gastro-enterostomy and the marked point 100 cm proximal to the ileocoecal junction.

After final stapling between the gastro-enterostomy and the entero-enterostomy the alimentary limb is measured using a set 5 cm marking on a babcock clamp.

#### Standard laparoscopic Roux-en-Y gastric bypass

Technique (linear gastro-enterostomy stapling technique):

Most steps are the same as mentioned for VLRL-RYGB. The only difference is the lengths described in the steps of creating the entero-enterostomy and measuring common channel instead of the alimentary limb.

After creation of the gastro-enterostomy, 60 cm from the point of Treitz, 150 cm is measured distal from the gastro-enterostomy and an entero-enterostomy is created between this point and the small intestine just proximal from the gastro-enterostomy.

After final stapling between the gastro-enterostomy and the entero-enterostomy the common channel is measured using a set 5 cm marking on a babcock clamp.

Technique (circular gastro-enterostomy stapling technique):

Most steps of this procedure are similar to the linear stapling technique. The technique differs in the creation of the gastro-enterostomy and the creation of the entero-enterostomy. Furthermore, in the circular technique the entero-enterostomy is created first.

In both groups and techniques the following is applicable:

Petersen’s space and other internal hernias can be closed by laparoscopic suturing or tackers and an abdominal silicone drain may be left behind at the surgeons’ preference. Staple line bleeding is controlled by electronic scissors, clips, or sutures. Next, the trocars are removed under sight and the skin is closed.

Antithrombosis prophylaxes and a fluid diet are continued for a minimum of two weeks postoperatively.

### Sample size determination

The sample size calculation is based on a superiority design, assuming that VLRL-RYGB is superior at 1 year follow-up when compared to standard LRYGB.

H0: mean % EWL at 1 year (standard LRYGB) = mean % EWL at 1 year (VLRL-RYGB)

H1: mean % EWL at 1 year (standard LRYGB) ≠ mean % EWL at 1 year (VLRL-RYGB)

To be able to reject the null hypothesis that mean % EWL at 1 year follow-up after standard LRYGB treatment equals mean % EWL at 1 year follow-up after VLRL-RYGB, at least 2x210 patients need to be included in the analysis. (Mean (SD) % EWL is 69.7 % (25.88) after VLRL-RYGB [19]; and 62.60 (25.88) after standard LRYGB; power = 80 %, randomization ratio 1:1). Considering a dropout rate of 5 %, the number of patients that need to be included is estimated to be 2x210/.95 = 2x221.1 = 444 (rounded up for equal number of patients per group).

The % EWL data of standard LRYGB are obtained from the meta analysis of Garb et al. [23]. The SD of VLRL-RYGB which currently is unreported is assumed to be equal to the SD of standard LRYGB.

### Study procedure

Data will be recorded prospectively on designated case record forms. All data are stored in a database, which is managed by the principal investigator. All adverse events will be investigated by a Data Safety Monitoring Board.

### Ethics approval

This study will be conducted in accordance to the standards of Good Clinical Practice, in agreement with the Declaration of Helsinki (latest amendment), Dutch law in general and with the W.M.O. in particular.

This trial has been approved by the regional medical ethical committee; Toetsingscommissie Wetenschappelijk Onderzoek Rotterdam e.o. (TWOR) at Maasstad Hospital Rotterdam,The Netherlands.

## Discussion

As mentioned in the background section, there are currently no randomized controlled trials addressing the role of the common channel in the treatment of the morbidly obese patient. Savassi et al. [24] addressed this issue by evaluating the length of the common channel in 100 patients that underwent LRYGB. They found a negative correlation (though weak) between weight loss and common limb length. Average common limb length was approximately 500 cm (range 268–829 cm) in patients receiving a standardized 110 cm AL and variable BL (average 56 cm, range 30–105 cm). They concluded that the common channel length does not play a role in weight loss for proximal (or short limbs) LRYGB but the variability in jejunoileal and common channel length should be considered when making modifications in the AL and BL.

Hernandez-Martinez et al. [25] performed LRYGB with a fixed common channel length of 230 cm in a series of 565 patients. After measuring the entire small bowel a length of 230 cm common channel was standardized and the rest of the small intestine was distributed as 60 % AL and 40 % BL. They selected 230 cm based on the assumption that this is the normal length of small bowel in adult non-obese persons. They found promising results in achieving long lasting EWL of over 70 % during 8-year follow-up with few metabolic complications.

In Scopinaro’s biliopancreatic diversion, distal gastrectomy is performed creating a 200–500 ml gastric pouch, with a 200-cm AL, a common channel of 50 cm, and a BL of 3–4 metres. Although this approach seems to be superior to RYGB for long term weight loss in super-obese patients [16, 26–29], high rates of metabolic complications are a concern [16, 28, 30, 31].

We hypothesize that our proposed VLRL-RYGB will provide for improved results concerning % EWL with an acceptable rate of (metabolic) complications in accordance with the results described by Thurnheer et al. [19] and Kalafarentzos et al. [17].

Our main point of interest is to determine if the VLRL-RYGB is a superior alternative to standard LRYGB in terms of percentage excess weight loss and to put more focus on the role of the common channel. Therefore we will perform this randomized controlled trial comparing both techniques, with % EWL as a primary outcome.

## Abbreviations

AE: Adverse event
AL: Alimentary limb
BAROS: Bariatric analysis and reporting outcome measures
BL: Biliopancreatic limb
BMI: Body mass Index (length/ weight^2^)
CC: Common channel
CCMO: Central Committee on Research Involving Human Subjects
DM2: Type 2 diabetes mellitus
LSG: Laparoscopic sleeve gastrectomy
LRYGB: Laparoscopic Roux-en-Y gastric bypass
METC: Medical research ethics committee (MREC); in Dutch: Medisch Ethische Toetsings Commissie (METC)
OSAS: Obstructive sleep apnea syndrome
%EWL: Percentage Extra Weight Loss (is calculated by using the formula EW = AW-IW (actual weight- ideal weight), IW = 22 × L^2^ (L = length in meters)
QOL: Quality of life
RCT: Randomized controlled trials
SAE: Serious adverse event
SF 36: Short Form 36
VLRL-RYGB: Very long Roux limb gastric bypass
WHO: The World Health Organization
